# Crystal structure and theoretical study of (2*E*)-1-[4-hy­droxy-3-(morpholin-4-ylmeth­yl)phen­yl]-3-(thio­phen-2-yl)prop-2-en-1-one

**DOI:** 10.1107/S2056989018008459

**Published:** 2018-06-12

**Authors:** Fatma Yesilyurt, Abdullah Aydin, Halise Inci Gul, Mehmet Akkurt, Nefise Dilek Ozcelik

**Affiliations:** a Department of Pharmaceutical Chemistry, Faculty of Pharmacy, Atatürk University, 25240 Erzurum, Turkey; b Department of Mathematics and Science Education, Faculty of Education, Kastamonu University, 37200 Kastamonu, Turkey; c Department of Physics, Faculty of Sciences, Erciyes University, 38039 Kayseri, Turkey; d Department of Physics, Faculty of Arts and Sciences, Aksaray University, 68100 Aksaray, Turkey

**Keywords:** crystal structure, theoretical study, quantum-chemical calculation, chalcones, Mannich bases, *HOMO*, *LUMO*

## Abstract

In the title compound, C_18_H_19_NO_3_S, the morpholine ring adopts a chair conformation. The thio­phene ring forms dihedral angles of 26.04 (9) and 74.07 (10)° with the benzene ring and the mean plane of the morpholine ring, respectively. The mol­ecular conformation is stabilized by an O—H⋯N hydrogen bond. In the crystal, mol­ecules are connected through C—H⋯O hydrogen bonds, forming wave-like layers parallel to the *ab* plane, which are further linked into a three-dimensional network by C—H⋯π inter­actions involving the benzene rings and the methyl­ene H atoms of the morpholine rings.

## Abstract 2

The mol­ecular conformation is stabilized by an intra­molecular O—H⋯N hydrogen bond. In the crystal, mol­ecules are linked by C—H⋯O hydrogen bonds, forming wave-like layers. C—H⋯π inter­actions involving the benzene rings and the methyl­ene hydrogen atoms of the morpholine rings are observed between the layers.

## Chemical context   

Chalcones, *viz* 1,3-diaryl-2-propene-1-ones, are major component of many natural products as well as important precursors for many synthetic manipulations (Das *et al.*, 2006 ▸; Yerdelen *et al.*, 2015 ▸; Gul *et al.*, 2009 ▸). Chalcones and their synthetic analogues display a wide range of biological activities such as anti­cancer, anti­malarial, anti­bacterial, anti-inflammatory, anti­fungal, anti­oxidant, anti-*HIV*, anti­protozoal, and carbonic anhydrase inhibiting activities (Das *et al.*, 2006 ▸; Yerdelen *et al.*, 2015 ▸; Gul *et al.*, 2007 ▸, 2009 ▸; Bilginer *et al.*, 2013 ▸, 2014 ▸; Yamali *et al.*, 2016 ▸; Singh *et al.*, 2014 ▸).
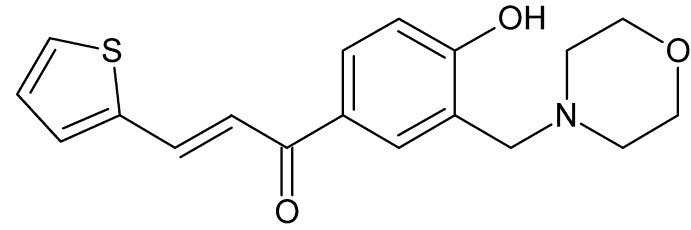


Mannich bases are an important class of compounds in medicinal chemistry. The Mannich reaction can be considered as a substitution reaction of a suitable compound in which one or more amino­methyl­ation processes happen, depending on the nature of the reactants. The biological activities of Mannich bases may result from their chemical structures or from the production of α,β-unsaturated ketone moieties (Roman, 2015 ▸). The title compound was designed with the expectation of observing an increased bioactivity or cytotoxicity in a mol­ecule including both chalcone and Mannich base pharmakophores.

## Structural commentary   

**Figure 1:**
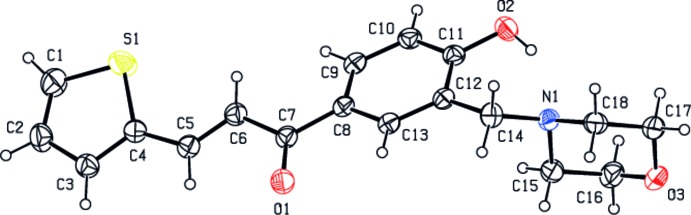
The mol­ecular structure of the title compound with displacement ellipsoids drawn at the 30% probability level

In the title compound (Fig. 1 ▸), the morpholine ring (N1/O3/C15–C18) adopts a chair conformation with puckering parameters *Q*_T_ = 0.5776 (18) Å, θ = 0.00 (19)°, φ = 308 (12)°. The benzene ring (C8–C13) forms dihedral angles of 26.04 (9) and 79.95 (8)° with the thio­phene ring (S1/C1–C4) and the mean plane of the morpholine ring, respectively. The values of all bond lengths and angles in the title compound are unexceptional. The mol­ecular conformation is enforced by an intra­molecular O—H⋯N hydrogen bond (Table 1 ▸).

## Supra­molecular features   

**Figure 2:**
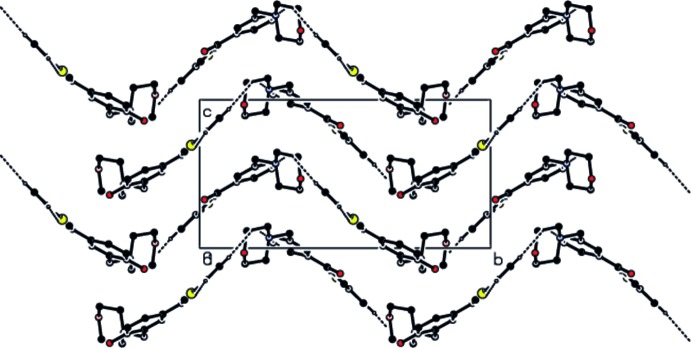
The mol­ecular packing of the title compound viewed down the *a* axis. Hydrogen bonds are shown as dashed lines.

In the crystal, mol­ecules are linked by inter­molecular C—H⋯O hydrogen bonds, forming wave-like layers parallel to the *ab* plane (Table 1 ▸, Fig. 2 ▸). C—H⋯π inter­actions are observed between the benzene rings and the methyl­ene hydrogen atoms of the morpholine rings in adjacent layers, forming a three-dimensional network.

## Database survey   

A search of the Cambridge Structural Database (Version 5.39, update May 2018; Groom *et al.*, 2016 ▸) for the 2-(morph­olino­meth­yl)phenol substructure yielded two hits, namely BOPMEY (Fun *et al.*, 1999 ▸) and IHUBIW (Xie *et al.*, 2003 ▸). In both compounds, the amine N atoms of the morpholine rings and the hy­droxy groups of the phenol fragments are engaged in intra­molecular hydrogen bonds.

## Theoretical calculations   

**Figure 3:**
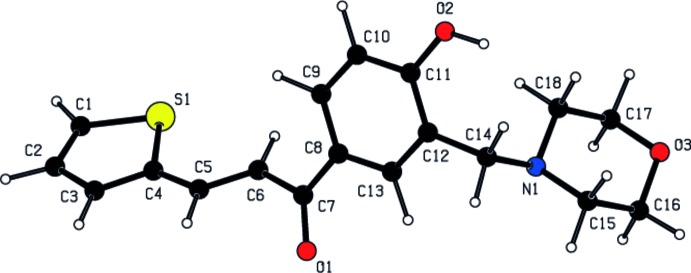
Spatial view of the title compound calculated using the *CNDO* method.

A quantum-chemical calculation was performed using the *CNDO* (Complete Neglect of Differential Overlap*;* Pople & Beveridge, 1970 ▸) approximation. A view of the calculated mol­ecule is shown in Fig. 3 ▸. The charges at atoms S1, O1, O2, O3 and N1 are −0.049, −0.336, −0.271, −0.224 and −0.145 e^−^, respectively. The calculated dipole moment of the title mol­ecule is *ca* 2.881 Debye. The *HOMO* and *LUMO* energy levels are −10.3681 and 1.4009 eV, respectively.

**Figure 4:**
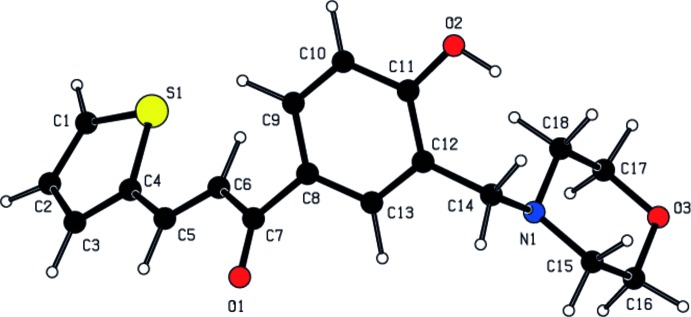
Spatial view of the title compound calculated using the *PM3* method

In addition, the geometrical optimization calculations of the title compound were performed using the *PM3* (Parameterized Model number 3) method (Stewart, 1989**a* ▸,b* ▸) in *WinMopac7.2*. A view of the mol­ecule calculated with *PM3* is shown in Fig. 4 ▸. The net charges at atoms S1, O1, O2, O3 and N1 are 0.321, −0.230, −0.260, −0.321 and −0.070 e^−^, respectively. The calculated dipole moment of the title mol­ecule is *ca* 1.176 Debye. The *HOMO* and *LUMO* energy levels are −0.1724 and 0.0829 eV, respectively. These calculations were performed assuming the mol­ecule to be isolated and in an absolute vacuum. A comparison between experimental and calculated bond lengths (r.m.s. deviations of 0.029 and 0.016 Å for *CNDO* and *PM3*, respectively) and angles (r.m.s. deviations of 1.601 and 1.915° for *CNDO* and *PM3*, respectively) is given in Table 2 ▸. The *PM3* method gave the lowest values for *HOMO*, *LUMO* and dipole moments.

## Synthesis and crystallization   

A mixture of paraformaldehyde (0.13 g, 4.3 mmol) and morpholine (0.37 g, 4.3 mmol) in aceto­nitrile (5 ml) was refluxed at 353 K for 30 min. A solution of a suitable chalcone in aceto­nitrile (25 ml), [1-(4-hy­droxy­phen­yl)-3-(thio­phene-2-yl)-2-propene-1-one (1 g, 4.3 mmol)], was added into the reaction flask under continuous heating. The reaction progress was monitored by TLC. The reaction stopped after 8 h when the chalcone compound was consumed in the reaction medium, and the solvent was removed under vacuum. The residue was purified by column chromotography (SiO_2_; CHCl_3_: MeOH 9:1 *v*/*v*). Yield 32%, m.p. 424–426 K. Crystals suitable for X-ray analysis were obtained by slow evaporation of a methanol solution.

## Refinement details   

Crystal data, data collection and structure refinement details are summarized in Table 3 ▸. C-bound H atoms were placed in calculated positions with C—H = 0.93–0.97 Å and refined using a riding model with *U*_iso_(H) = 1.2*U*_eq_(C). The hy­droxy H atom was found in a difference-Fourier map and refined with *U*_iso_(H) = 1.5*U*_eq_(O). 15 outliers (5 4 6, 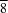 14 1, 5 3 2, 3 4 2, 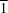 3 1, 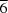 16 4, 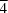 11 1, 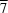 7 9, 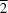 11 1, 2 2 10, 0 5 12, 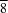 13 1, 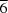 13 3, 0 15 4, 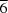 17 4) were omitted in the final cycles of refinement.

## Supplementary Material

Crystal structure: contains datablock(s) global, I. DOI: 10.1107/S2056989018008459/rz5238sup1.cif

Structure factors: contains datablock(s) I. DOI: 10.1107/S2056989018008459/rz5238Isup4.hkl

Supporting information file. DOI: 10.1107/S2056989018008459/rz5238Isup3.cml

CCDC reference: 1848116

Additional supporting information:  crystallographic information; 3D view; checkCIF report

## Figures and Tables

**Table 1:** Hydrogen-bond geometry (Å, °) *Cg*1 is the centroid of the C8–C13 benzene ring.

*D*—H⋯*A*	*D*—H	H⋯*A*	*D*⋯*A*	*D*—H⋯*A*
O2—H1*O*⋯N1	0.83 (2)	1.94 (2)	2.6834 (18)	149 (3)
C1—H1⋯O1^i^	0.93	2.38	3.249 (2)	156
C2—H2⋯O2^ii^	0.93	2.57	3.417 (2)	152
C16—H16*A*⋯*Cg*1^iii^	0.97	2.88	3.789 (2)	157
C18—H18*B*⋯*Cg*1^iv^	0.97	2.70	3.6010 (18)	154

Symmetry codes: (i) 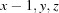; (ii) 
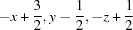
; (iii) 
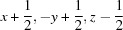
; (iv) 
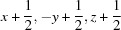
.

**Table 2:** Comparison of experimental (X-ray), theoretical (*CNDO* and *PM3*) parameters (Å, °) of the title compound

Bond/Angle	X-ray	*CNDO*	PM3
S1—C1	1.705 (2)	1.7663	1.7194
S1—C4	1.720 (2)	1.7758	1.7449
O1—C7	1.224 (2)	1.2143	1.2196
O2—C11	1.354 (2)	1.3565	1.3663
O3—C16	1.419 (2)	1.4208	1.4149
O3—C17	1.422 (2)	1.4209	1.4153
N1—C14	1.472 (2)	1.4606	1.4916
N1—C15	1.469 (2)	1.4573	1.4914
N1—C18	1.469 (2)	1.4567	1.4906
			
C1—S1—C4	92.20 (9)	88.91	91.38
C16—O3—C17	109.29 (13)	110.44	112.79
C14—N1—C15	111.86 (13)	111.15	112.06
C14—N1—C18	110.61 (13)	111.92	112.86
C15—N1—C18	109.09 (13)	109.64	111.62
S1—C1—C2	111.75 (15)	111.11	112.58
S1—C4—C5	123.58 (12)	126.03	125.76
S1—C4—C3	109.79 (12)	109.88	111.11
O1—C7—C6	120.42 (14)	119.03	122.82
O1—C7—C8	119.90 (14)	123.49	121.52
O2—C11—C10	118.59 (14)	119.87	115.22
O2—C11—C12	121.18 (14)	122.06	123.98
N1—C14—C12	112.14 (12)	112.35	111.21
N1—C15—C16	109.98 (14)	110.79	109.89
O3—C16—C15	111.42 (17)	109.89	112.44
O3—C17—C18	111.22 (14)	110.05	112.30
N1—C18—C17	109.80 (15)	110.73	110.02

**Table 3:** Experimental details

Crystal data	
Chemical formula	C_18_H_19_NO_3_S
*M*_r_	329.40
Crystal system, space group	Monoclinic, *P*2_1_/*n*
Temperature (K)	293
*a*, *b*, *c* (Å)	9.4939 (5), 18.5548 (10), 9.5068 (5)
β (°)	96.788 (3)
*V* (Å^3^)	1662.95 (15)
*Z*	4
Radiation type	Mo *K*α
μ (mm^−1^)	0.21
Crystal size (mm)	0.81 × 0.50 × 0.48

Data collection	
Diffractometer	Bruker APEXII CCD
Absorption correction	Multi-scan (*SADABS*; Bruker, 2007 ▸)
*T*_min_, *T*_max_	0.882, 0.905
No. of measured, independent and observed [*I* > 2σ(*I*)] reflections	33902, 4168, 3373
*R*_int_	0.033
(sin θ/λ)_max_ (Å^−1^)	0.670

Refinement	
*R*[*F* ^2^ > 2σ(*F* ^2^)], *wR*(*F* ^2^), *S*	0.050, 0.138, 1.03
No. of reflections	4168
No. of parameters	211
No. of restraints	1
H-atom treatment	H atoms treated by a mixture of independent and constrained refinement
Δρ_max_, Δρ_min_ (e Å^−3^)	0.32, −0.25

Computer programs: *APEX2* and *SAINT* (Bruker, 2007 ▸), *SHELXS2014* (Sheldrick, 2008 ▸), *SHELXL2014* (Sheldrick, 2008 ▸), *ORTEP-3 for Windows* (Farrugia, 2012 ▸) and *PLATON* (Spek, 2009 ▸).

## supplementary crystallographic information

### Crystal data

**Table:**

C_18_H_19_NO_3_S	*F*(000) = 696
*M**_r_* = 329.40	*D*_x_ = 1.316 Mg m^−^^3^
Monoclinic, *P*2_1_/*n*	Mo *K*α radiation, λ = 0.71073 Å
*a* = 9.4939 (5) Å	Cell parameters from 9868 reflections
*b* = 18.5548 (10) Å	θ = 2.2–28.4°
*c* = 9.5068 (5) Å	µ = 0.21 mm^−^^1^
β = 96.788 (3)°	*T* = 293 K
*V* = 1662.95 (15) Å^3^	Prism, colourless
*Z* = 4	0.81 × 0.50 × 0.48 mm

### Data collection

**Table:**

Bruker APEXII CCD diffractometer	4168 independent reflections
Radiation source: sealed tube	3373 reflections with *I* > 2σ(*I*)
Graphite monochromator	*R*_int_ = 0.033
φ and ω scans	θ_max_ = 28.5°, θ_min_ = 2.2°
Absorption correction: multi-scan (SADABS; Bruker, 2007)	*h* = −12→12
*T*_min_ = 0.882, *T*_max_ = 0.905	*k* = −24→24
33902 measured reflections	*l* = −12→12

### Refinement

**Table:**

Refinement on *F*^2^	1 restraint
Least-squares matrix: full	Hydrogen site location: mixed
*R*[*F*^2^ > 2σ(*F*^2^)] = 0.050	H atoms treated by a mixture of independent and constrained refinement
*wR*(*F*^2^) = 0.138	*w* = 1/[σ^2^(*F*_o_^2^) + (0.0679*P*)^2^ + 0.4859*P*] where *P* = (*F*_o_^2^ + 2*F*_c_^2^)/3
*S* = 1.03	(Δ/σ)_max_ < 0.001
4168 reflections	Δρ_max_ = 0.32 e Å^−^^3^
211 parameters	Δρ_min_ = −0.25 e Å^−^^3^

### Special details

**Table:**

Geometry. All esds (except the esd in the dihedral angle between two l.s. planes) are estimated using the full covariance matrix. The cell esds are taken into account individually in the estimation of esds in distances, angles and torsion angles; correlations between esds in cell parameters are only used when they are defined by crystal symmetry. An approximate (isotropic) treatment of cell esds is used for estimating esds involving l.s. planes.

### Fractional atomic coordinates and isotropic or equivalent isotropic displacement parameters (Å^2^)

**Table:**

	*x*	*y*	*z*	*U*_iso_*/*U*_eq_	
C1	0.3316 (2)	−0.02309 (12)	0.2121 (3)	0.0720 (6)	
H1	0.235465	−0.019863	0.221873	0.086*	
C2	0.3860 (2)	−0.06817 (11)	0.1231 (2)	0.0623 (5)	
H2	0.331381	−0.099790	0.063433	0.075*	
C3	0.53423 (18)	−0.06285 (9)	0.12897 (19)	0.0522 (4)	
H3	0.587701	−0.090438	0.073164	0.063*	
C4	0.59198 (16)	−0.01301 (8)	0.22504 (17)	0.0441 (3)	
C5	0.74016 (16)	0.00373 (8)	0.25820 (17)	0.0447 (3)	
H5	0.802258	−0.022598	0.209531	0.054*	
C6	0.79903 (17)	0.05204 (9)	0.34953 (18)	0.0465 (4)	
H6	0.741687	0.080254	0.400249	0.056*	
C7	0.95402 (16)	0.06168 (8)	0.37183 (17)	0.0433 (3)	
C8	1.01520 (15)	0.12698 (8)	0.44638 (15)	0.0398 (3)	
C9	0.93436 (16)	0.18710 (8)	0.47309 (16)	0.0435 (3)	
H9	0.836671	0.186378	0.448048	0.052*	
C10	0.99807 (17)	0.24766 (8)	0.53638 (17)	0.0456 (3)	
H10	0.943157	0.287658	0.552522	0.055*	
C11	1.14360 (16)	0.24932 (8)	0.57615 (15)	0.0410 (3)	
C12	1.22777 (15)	0.18990 (8)	0.54966 (15)	0.0390 (3)	
C13	1.16227 (15)	0.13017 (8)	0.48509 (15)	0.0397 (3)	
H13	1.217489	0.090658	0.466589	0.048*	
C14	1.38542 (17)	0.19118 (9)	0.59817 (18)	0.0473 (4)	
H14A	1.401057	0.181264	0.698985	0.057*	
H14B	1.431423	0.153440	0.549728	0.057*	
C15	1.45567 (19)	0.27260 (10)	0.41787 (17)	0.0533 (4)	
H15A	1.360309	0.272216	0.368303	0.064*	
H15B	1.508851	0.233930	0.380183	0.064*	
C16	1.5255 (2)	0.34382 (12)	0.3944 (2)	0.0645 (5)	
H16A	1.529884	0.350654	0.293866	0.077*	
H16B	1.468902	0.382512	0.427116	0.077*	
C17	1.65806 (19)	0.33755 (12)	0.6150 (2)	0.0603 (5)	
H17A	1.601485	0.375827	0.649325	0.072*	
H17B	1.752902	0.340669	0.665265	0.072*	
C18	1.59421 (16)	0.26603 (10)	0.64480 (18)	0.0507 (4)	
H18A	1.652343	0.227514	0.613763	0.061*	
H18B	1.591062	0.260745	0.745894	0.061*	
N1	1.45003 (13)	0.26102 (7)	0.56999 (13)	0.0425 (3)	
O1	1.03248 (13)	0.01683 (7)	0.32762 (15)	0.0605 (3)	
O2	1.20139 (14)	0.30917 (7)	0.64087 (14)	0.0548 (3)	
O3	1.66467 (14)	0.34704 (8)	0.46745 (15)	0.0686 (4)	
S1	0.45988 (5)	0.02779 (3)	0.30547 (7)	0.0781 (2)	
H1O	1.2875 (19)	0.3090 (18)	0.633 (3)	0.117*	

### Atomic displacement parameters (Å^2^)

**Table:**

	*U*^11^	*U*^22^	*U*^33^	*U*^12^	*U*^13^	*U*^23^
C1	0.0407 (9)	0.0815 (14)	0.0932 (15)	0.0020 (9)	0.0062 (9)	−0.0139 (12)
C2	0.0505 (10)	0.0600 (11)	0.0743 (13)	−0.0078 (8)	−0.0017 (9)	−0.0083 (9)
C3	0.0508 (9)	0.0475 (9)	0.0585 (10)	0.0007 (7)	0.0072 (7)	−0.0053 (7)
C4	0.0416 (8)	0.0406 (8)	0.0499 (8)	0.0049 (6)	0.0046 (6)	0.0006 (6)
C5	0.0417 (8)	0.0414 (7)	0.0515 (8)	0.0031 (6)	0.0073 (6)	0.0028 (6)
C6	0.0419 (8)	0.0443 (8)	0.0538 (9)	0.0012 (6)	0.0084 (7)	−0.0019 (7)
C7	0.0426 (8)	0.0413 (8)	0.0470 (8)	−0.0013 (6)	0.0094 (6)	0.0031 (6)
C8	0.0394 (7)	0.0408 (7)	0.0405 (7)	−0.0009 (6)	0.0098 (6)	0.0044 (6)
C9	0.0370 (7)	0.0476 (8)	0.0472 (8)	0.0017 (6)	0.0102 (6)	0.0029 (6)
C10	0.0451 (8)	0.0430 (8)	0.0505 (9)	0.0060 (6)	0.0132 (7)	−0.0018 (6)
C11	0.0468 (8)	0.0414 (7)	0.0359 (7)	−0.0002 (6)	0.0086 (6)	0.0015 (6)
C12	0.0388 (7)	0.0417 (7)	0.0367 (7)	0.0007 (6)	0.0052 (6)	0.0066 (6)
C13	0.0401 (7)	0.0372 (7)	0.0427 (7)	0.0034 (6)	0.0090 (6)	0.0049 (6)
C14	0.0427 (8)	0.0468 (8)	0.0509 (9)	0.0011 (6)	−0.0012 (6)	0.0040 (7)
C15	0.0532 (9)	0.0672 (11)	0.0389 (8)	−0.0119 (8)	0.0035 (7)	−0.0032 (7)
C16	0.0651 (11)	0.0769 (13)	0.0532 (10)	−0.0191 (10)	0.0138 (8)	0.0029 (9)
C17	0.0433 (9)	0.0802 (13)	0.0587 (10)	−0.0145 (8)	0.0114 (7)	−0.0211 (9)
C18	0.0379 (8)	0.0673 (11)	0.0461 (8)	0.0016 (7)	0.0016 (6)	−0.0121 (7)
N1	0.0376 (6)	0.0507 (7)	0.0388 (6)	−0.0043 (5)	0.0032 (5)	−0.0020 (5)
O1	0.0456 (6)	0.0539 (7)	0.0837 (9)	0.0002 (5)	0.0151 (6)	−0.0174 (6)
O2	0.0554 (7)	0.0482 (6)	0.0605 (7)	−0.0024 (5)	0.0059 (6)	−0.0127 (5)
O3	0.0540 (7)	0.0911 (10)	0.0646 (8)	−0.0251 (7)	0.0234 (6)	−0.0133 (7)
S1	0.0495 (3)	0.0889 (4)	0.0965 (4)	0.0074 (2)	0.0111 (3)	−0.0427 (3)

### Geometric parameters (Å, º)

**Table:**

C1—C2	1.336 (3)	C11—C12	1.402 (2)
C1—S1	1.705 (2)	C12—C13	1.379 (2)
C1—H1	0.9300	C12—C14	1.513 (2)
C2—C3	1.406 (2)	C13—H13	0.9300
C2—H2	0.9300	C14—N1	1.472 (2)
C3—C4	1.368 (2)	C14—H14A	0.9700
C3—H3	0.9300	C14—H14B	0.9700
C4—C5	1.439 (2)	C15—N1	1.469 (2)
C4—S1	1.7197 (16)	C15—C16	1.507 (3)
C5—C6	1.325 (2)	C15—H15A	0.9700
C5—H5	0.9300	C15—H15B	0.9700
C6—C7	1.473 (2)	C16—O3	1.419 (2)
C6—H6	0.9300	C16—H16A	0.9700
C7—O1	1.2239 (19)	C16—H16B	0.9700
C7—C8	1.487 (2)	C17—O3	1.422 (2)
C8—C9	1.394 (2)	C17—C18	1.500 (3)
C8—C13	1.403 (2)	C17—H17A	0.9700
C9—C10	1.380 (2)	C17—H17B	0.9700
C9—H9	0.9300	C18—N1	1.4691 (19)
C10—C11	1.389 (2)	C18—H18A	0.9700
C10—H10	0.9300	C18—H18B	0.9700
C11—O2	1.3538 (19)	O2—H1O	0.830 (18)
			
C2—C1—S1	111.75 (15)	C8—C13—H13	118.9
C2—C1—H1	124.1	N1—C14—C12	112.14 (12)
S1—C1—H1	124.1	N1—C14—H14A	109.2
C1—C2—C3	113.02 (18)	C12—C14—H14A	109.2
C1—C2—H2	123.5	N1—C14—H14B	109.2
C3—C2—H2	123.5	C12—C14—H14B	109.2
C4—C3—C2	113.24 (16)	H14A—C14—H14B	107.9
C4—C3—H3	123.4	N1—C15—C16	109.98 (14)
C2—C3—H3	123.4	N1—C15—H15A	109.7
C3—C4—C5	126.63 (15)	C16—C15—H15A	109.7
C3—C4—S1	109.79 (12)	N1—C15—H15B	109.7
C5—C4—S1	123.58 (12)	C16—C15—H15B	109.7
C6—C5—C4	127.99 (15)	H15A—C15—H15B	108.2
C6—C5—H5	116.0	O3—C16—C15	111.42 (17)
C4—C5—H5	116.0	O3—C16—H16A	109.3
C5—C6—C7	120.90 (15)	C15—C16—H16A	109.3
C5—C6—H6	119.6	O3—C16—H16B	109.3
C7—C6—H6	119.6	C15—C16—H16B	109.3
O1—C7—C6	120.42 (14)	H16A—C16—H16B	108.0
O1—C7—C8	119.90 (14)	O3—C17—C18	111.22 (14)
C6—C7—C8	119.67 (13)	O3—C17—H17A	109.4
C9—C8—C13	118.09 (14)	C18—C17—H17A	109.4
C9—C8—C7	123.12 (14)	O3—C17—H17B	109.4
C13—C8—C7	118.69 (13)	C18—C17—H17B	109.4
C10—C9—C8	120.57 (14)	H17A—C17—H17B	108.0
C10—C9—H9	119.7	N1—C18—C17	109.80 (15)
C8—C9—H9	119.7	N1—C18—H18A	109.7
C9—C10—C11	120.48 (14)	C17—C18—H18A	109.7
C9—C10—H10	119.8	N1—C18—H18B	109.7
C11—C10—H10	119.8	C17—C18—H18B	109.7
O2—C11—C10	118.59 (14)	H18A—C18—H18B	108.2
O2—C11—C12	121.18 (14)	C18—N1—C15	109.09 (13)
C10—C11—C12	120.23 (14)	C18—N1—C14	110.61 (13)
C13—C12—C11	118.39 (13)	C15—N1—C14	111.86 (13)
C13—C12—C14	121.74 (13)	C11—O2—H1O	108 (2)
C11—C12—C14	119.80 (13)	C16—O3—C17	109.29 (13)
C12—C13—C8	122.22 (13)	C1—S1—C4	92.20 (9)
C12—C13—H13	118.9		
			
S1—C1—C2—C3	0.3 (3)	C10—C11—C12—C14	−177.78 (14)
C1—C2—C3—C4	0.4 (3)	C11—C12—C13—C8	−0.4 (2)
C2—C3—C4—C5	178.40 (16)	C14—C12—C13—C8	176.60 (13)
C2—C3—C4—S1	−0.9 (2)	C9—C8—C13—C12	0.9 (2)
C3—C4—C5—C6	179.33 (18)	C7—C8—C13—C12	177.50 (13)
S1—C4—C5—C6	−1.5 (3)	C13—C12—C14—N1	140.07 (14)
C4—C5—C6—C7	179.26 (15)	C11—C12—C14—N1	−42.94 (19)
C5—C6—C7—O1	−13.9 (3)	N1—C15—C16—O3	58.3 (2)
C5—C6—C7—C8	165.08 (15)	O3—C17—C18—N1	−59.52 (19)
O1—C7—C8—C9	166.04 (15)	C17—C18—N1—C15	56.66 (17)
C6—C7—C8—C9	−12.9 (2)	C17—C18—N1—C14	−179.90 (13)
O1—C7—C8—C13	−10.4 (2)	C16—C15—N1—C18	−55.98 (19)
C6—C7—C8—C13	170.68 (14)	C16—C15—N1—C14	−178.68 (15)
C13—C8—C9—C10	−0.3 (2)	C12—C14—N1—C18	167.76 (13)
C7—C8—C9—C10	−176.70 (14)	C12—C14—N1—C15	−70.41 (17)
C8—C9—C10—C11	−0.8 (2)	C15—C16—O3—C17	−59.5 (2)
C9—C10—C11—O2	−178.50 (14)	C18—C17—O3—C16	60.1 (2)
C9—C10—C11—C12	1.3 (2)	C2—C1—S1—C4	−0.7 (2)
O2—C11—C12—C13	179.13 (13)	C3—C4—S1—C1	0.90 (15)
C10—C11—C12—C13	−0.7 (2)	C5—C4—S1—C1	−178.40 (16)
O2—C11—C12—C14	2.0 (2)		

### Hydrogen-bond geometry (Å, º)

*Cg*1 is the centroid of the C8–C13 benzene ring.

**Table:**

*D*—H···*A*	*D*—H	H···*A*	*D*···*A*	*D*—H···*A*
O2—H1*O*···N1	0.83 (2)	1.94 (2)	2.6834 (18)	149 (3)
C1—H1···O1^i^	0.93	2.38	3.249 (2)	156
C2—H2···O2^ii^	0.93	2.57	3.417 (2)	152
C5—H5···O1	0.93	2.45	2.786 (2)	101
C16—H16*A*···*Cg*1^iii^	0.97	2.88	3.789 (2)	157
C18—H18*B*···*Cg*1^iv^	0.97	2.70	3.6010 (18)	154

Symmetry codes: (i) *x*−1, *y*, *z*; (ii) −*x*+3/2, *y*−1/2, −*z*+1/2; (iii) *x*+1/2, −*y*+1/2, *z*−1/2; (iv) *x*+1/2, −*y*+1/2, *z*+1/2.
